# Recent Advances in Soft Matter

**DOI:** 10.3390/ma18071440

**Published:** 2025-03-25

**Authors:** Ingo Dierking

**Affiliations:** Department of Physics and Astronomy, University of Manchester, Oxford Road, Manchester M13 9PL, UK; ingo.dierking@manchester.ac.uk

As the current Section Editor for Soft Matter of *Materials*, I am delighted to be able to present a Special Issue of the journal: The 15th Anniversary of *Materials*—Recent Advances in Soft Matter.

Today, the highly interdisciplinary field of soft matter combines chemical synthesis, physico-chemical experiments and interpretation, physical theory and modelling of experimental data, instrumentation development, material sciences, biology and biochemistry, and medicine and healthcare, all the way to manufacturing and materials processing. Traditional fields such as polymers, colloids, gels, granular systems, and liquid crystals and foams are increasingly combined and overlap, incorporating medical and biochemical aspects. This leads to a plethora of additional application areas beyond plastics, foods and cosmetics, towards further modern aspects of soft matter like nanotechnology, photonics, soft robotics, and smart glass or sensors, yet without disregarding the traditional fundamental aspects of self-assembly, phase transitions or rheology.

Soft-matter materials are easily deformable due to their very small elastic constants when compared to solid-state materials. Thus, large deformations will easily occur with only very small external forces applied. While deformations in solid-state materials are of the order of a few atomic distances, soft-matter materials can show deformations of several hundred micrometres and larger for similar forces applied. Out of the many examples, one can, for instance, mention the spatial size of defects found in liquid crystals, which are easily seen in polarisation microscopy, while dislocations in solids are of the size of a few nanometres. Another aspect of soft matter is the fact that interesting physical behaviour occurs, i.e., intermolecular interactions, at energies comparable to room temperature ~kT. This implies that temperature plays a dominant role and materials react in a very sensitive way to fluctuations in external conditions. An example here would be the sensitivity for coagulation vs. peptization of a colloidal dispersion. At last, an often-observed phenomenon in soft matter is self-assembly and self-organisation, which leads to mesoscopic structures but also to complexity in dynamics and properties. Here, we can find examples in surfactant solutions where, at the critical micelle concentration, tens of individual surfactant molecules self-assemble into one micelle. At the same time, the surface tension strongly decreases, while the viscosity strongly increases. Yet the structural dynamics is rather complex, with surfactant molecules leaving and entering the micelle, which can even disappear completely, while a new micelle is formed elsewhere.

Taking a look at Figure 1, which depicts the number of annual publications mentioning the term “Soft Matter” over the last three decades, one could be tempted to say that the field experienced an explosive increase in interest in the five years following 2005 and has since matured into a field of research with a constant output in the last fifteen years. Nonetheless, as we know, soft materials have been around for much longer but, most likely, were only referred to as individual fields, like polymers, colloids, gels, surfactants, or liquid crystals. Only in this millennium have these sub-fields of soft matter largely influenced and fruitfully enriched each other, forming a synergy which makes this topic so timely, scientifically challenging and applicationally interesting.

For any reader who would like to acquire a first and general overview of experimental soft matter [1], its grand challenges [2] and a recently proposed roadmap [3] can be recommended, in which the directions that the field might be developing in the near and medium future are outlined.

One of the current fields that should be highlighted is the use of polymers, or, rather, elastomers. Already, here, one can realise that other fields besides polymers have enriched this field, namely liquid crystals, in the form of liquid crystal elastomers. These promise use for various applications, such as colour-changing films under application of deformations [4], but also the converse effect, light-induced deformations through incorporated azo-groups [5]. These materials can be printed [6]; fibres can be drawn and woven into fabrics [7] to act as actuators, power sources, or sensors triggered by light, solvents, vapour, electric or magnetic fields, or temperature, which promise impact in the general field of soft robotics [8]. The latter is not only often bio-inspired and realised via liquid crystal elastomers [9], but also via hydrogels [10], and materials can perform sensing and other duties via electric or magnetic stimulation towards soft intelligent robots [11].

Another strongly expanding field in the area of soft matter is currently found in biomaterials and healthcare, again exploiting the interdisciplinarity with other soft-matter aspects like gels [12], applications of robotics [13], colloids for drug delivery [14], biodegradable polymers in healthcare [15], or even viruses as model systems for colloidal and liquid crystal behaviour [16].

In the realm of classical colloids, these materials find novel applications, for example, as quantum materials [17] or in the preservation of art [18]. Yet the topic which has probably gained the most attention in recent years is that of driven or active (self-driven) soft matter [19]. This has found its way into metamaterials [20], biomimetics [21], and the field of collective behaviour [22], just to name a few topics. Active materials from soft matter have experienced such an explosive growth of interest that even a survey about reviews of the different subtopics was published a few weeks ago in 2025 [23].

Also, investigations of liquid crystals have moved away from the classic field of displays towards elastomers [24,25,26] (as already mentioned above), stimuli-responsive smart materials [27], chiral materials mimicking nature [28], and enhancing the field of photonics [29] by making materials tuneable through external stimuli. The latter aspect is also exploited for liquid crystal sensors for biomolecules, solvents or gases [30,31]. With respect to real applications to date, liquid crystal–polymer composites in the form of smart windows/smart glass have flourished considerably in the last few years [32,33,34,35].

In terms of fundamental science relating to soft matter, three main areas of recent interest should be pointed out: (i) advances in theory, simulation and modelling [36,37], (ii) the rise of artificial intelligence and machine learning [38,39], and (iii) mathematical descriptions of soft matter, in particular the field of topology [40,41]. But we should also not disregard the topics which have long been a classical aspect of soft materials, for example, at the interface to biology and biophysics, namely cells and vesicles. Also here, some very interesting developments have taken place, for example, in the use of vesicles for drug delivery [42]. These vesicles can even be derived from cells [43], or conversely, vesicles can be used in the development of protocells [44] or for cell-to-cell communication [45]. A field which will probably expand in the future has been outlined as optobiology [46], the use of living cells in optics and photonics.

From this certainly incomplete discussion of various soft-matter topics, it is clear that the field displays an enormous breadth and great potential for fundamental physics, synthetic chemistry, and the development of novel materials, but above all, for a plethora of potential applications from photonics to actuators, from sensors to soft robotics, and from biological functionality to healthcare products and engineering. The reader of this Special Issue will find that this breadth of soft-matter aspects is replicated in this book to celebrate 15 years of the journal *Materials* with a special focus on “Recent Advances in Soft Matter”. I have broadly grouped the published research papers and reviews into four categories to illustrate the diversity and breadth of the topic:

A: Liquid crystals and composite systems—covering subjects as diverse as multi-stable cholesteric LCs [47], ionic LCs as photonic materials [48], and a Voronoi description of polymer-modified LCs [49].

B: Soft polymers—reviewing stimuli-responsive smart polymers [50], and modelling rate-dependent adhesion [51].

C: Biomaterials—discussing silk fibroin-based biomedical soft materials [52], hybrid biopolymer films [53], and reverse micelle extraction from biomaterials [54].

D: Colloid and active matter—studying bacteria-based colloidal active matter and collective behaviour [55], and the synthesis of coated magnetic colloids [56].

With this, I would like to wish the journal a happy 15th anniversary and the reader some hopefully interesting, inspiring, diverse and varied reading on the aspects of soft-matter materials.

## Figures and Tables

**Figure 1:**
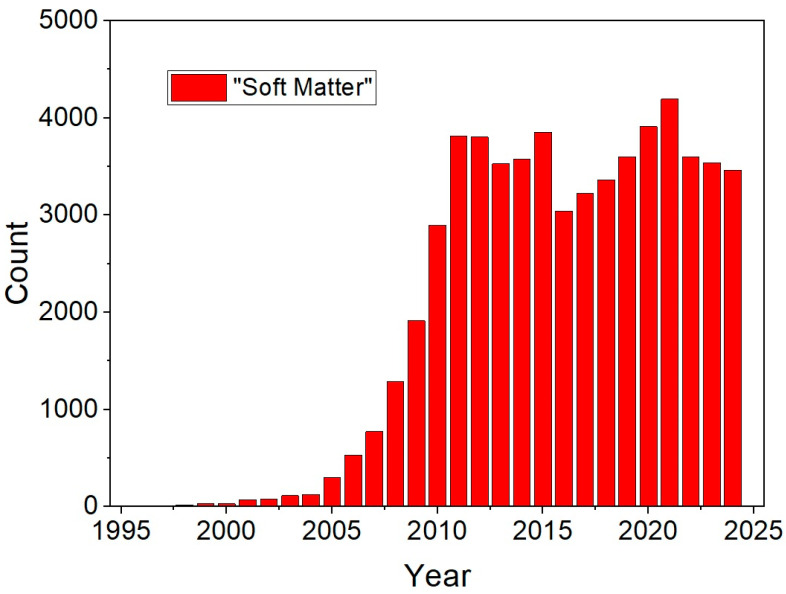
Number of publications in each year mentioning the term “Soft Matter” for the last three decades.
